# COVID-19 preparedness at health facilities and community service points serving people living with HIV in Sierra Leone

**DOI:** 10.1371/journal.pone.0250236

**Published:** 2021-04-15

**Authors:** Lauren E. Parmley, Kieran Hartsough, Oliver Eleeza, Akopon Bertin, Bockarie Sesay, Amon Njenga, Mame Toure, Ginika Egesimba, Haja Bah, Alex Bayoh, Abdulraheem Yakubu, Ellen A. B. Morrison, Susan Michaels-Strasser

**Affiliations:** 1 ICAP at Columbia University, New York, New York, United States of America; 2 ICAP at Columbia University, Freetown, Sierra Leone; National Institute for Infectious Diseases Lazzaro Spallanzani-IRCCS, ITALY

## Abstract

After a decade of civil war and the 2014–2016 West African Ebola outbreak, Sierra Leone now faces the COVID-19 pandemic with a fragile health system. As was demonstrated during Ebola, preparedness is key to limiting a health crisis’ spread and impact on health systems and ensuring continued care for vulnerable populations including people living with HIV (PLHIV). To assess COVID-19 preparedness and inform interventions to ensure continuity of HIV services at health facilities (HFs) and community service points (CSPs), we conducted site readiness assessments in Freetown, the epicenter of COVID-19 in Sierra Leone. Data were collected at nine high-volume HIV HFs and seven CSPs in April 2020, a month after COVID-19 was declared a pandemic. CSPs comprised three community drop-in centers providing HIV counseling and testing services as well as HIV prevention services (e.g., condoms and lubricants) for key and priority populations and four community-based support groups serving PLHIV. At the time of assessment, CSPs did not provide antiretroviral therapy (ART) but were considered potential sites for expansion of differentiated service delivery (DSD)—a client-centered approach to HIV care—in the context of COVID-19. Overall, 5/9 HFs had trained staff on use of personal protective equipment (PPE) and prevention of COVID-19 transmission. Most had access to masks (5/9) and gloves (7/9) for management of suspected/confirmed COVID-19 cases, and 4/9 HFs had triage procedures for isolation of suspected cases. Conversely, few CSPs had access to masks (2/7) or gloves (2/7) and no staff were trained on PPE use or COVID-19 transmission. 7/9 HFs had adequate ART stock for multi-month dispensing though few had procedures for (3/9) or had trained staff in providing DSD (2/9). Among CSPs where measures were applicable, 2/4 had procedures for DSD, 1/3 had staff trained on DSD and none had adequate ART stock. Identification of gaps in COVID-19 preparedness is a critical step in providing support for infection control and modified service delivery. Findings from this assessment highlight gaps in COVID-19 preparedness measures at sites supporting PLHIV in Sierra Leone and indicate CSPs may require intensive supervision and training to ensure HIV services are uninterrupted while minimizing COVID-19 risk, especially if used as sites to scale up DSD.

## Introduction

Five years on from the 2014–2016 West African Ebola outbreak which resulted in nearly 4,000 deaths in Sierra Leone [1], including 7% of deaths among the total health work force, the country now faces the COVID-19 pandemic [2, 3]. Ebola weakened an already fragile health system devastated by a decade of civil war, and contributed to Sierra Leone having one of the world’s most severe healthcare worker (HCW) shortages with most recent estimates of just.024 physicians and.319 nursing and midwifery personnel per 1000 population [4]. A key marker of a country’s health system, Sierra Leone has one of the highest maternal mortality ratios globally [5].

As was demonstrated during Ebola, preparedness is key to limiting a health crisis’ impact on health systems and ensuring continued care for vulnerable populations including people living with HIV (PLHIV) [6]. Disruptions in the provision of routine healthcare services, including those related to HIV can lead to adverse health outcomes, and, in the case of HIV, threaten to reduce progress achieved in the global HIV response. As Ebola overwhelmed health systems in West Africa, health facilities (HFs) in all affected countries interrupted or reduced HIV services including HIV testing and treatment [7, 8]. In Sierra Leone, the number of PLHIV on antiretroviral therapy (ART) declined during the peak of the Ebola outbreak, and correlations were found between districts most affected by Ebola and longer periods of decline in ART patients [9]. Among military personnel, a priority population in Sierra Leone, there was higher risk of interruption in continuity of treatment during the outbreak compared to pre-Ebola, and the largest increase in risk occurred during Ebola’s peak [10]. This evidence indicates geographic areas most affected by an epidemic may experience more severe HIV service disruption and continuity of HIV care may be hardest to ensure during the height of a health crisis.

COVID-19, the most recent global health threat, has upended healthcare systems in the world’s most resource-rich countries [11, 12], and the impact is anticipated to be more severe and lasting in low-resource countries, such as Sierra Leone [13]. Recent modeling of potential effects caused by COVID-19 suggests a six-month disruption of supply in ART for 50% of PLHIV would increase HIV-related deaths and mother-to-child transmission nearly two-fold in just one year in sub-Saharan Africa [14, 15]. Limited resources and institutional vulnerabilities have already contributed to substantial gaps in health metrics for HIV in Sierra Leone; only 49% of adults aged 15–49 years living with HIV in Sierra Leone are aware of their status, less than half of PLHIV are on ART, and a quarter are virally suppressed [16, 17]. These outcomes are far below the global UNAIDS Fast-Track targets to end the AIDS epidemic by 2030 [18] and may be worsened by COVID-19-related disruptions.

### COVID-19 in Africa and Sierra Leone

Beyond concerns of HIV-related mortality and new infections, many African countries are ill prepared to manage and respond to the COVID-19 pandemic. In Africa, where almost 20% of the world’s population resides (approximately 1.2 billion people), estimates indicate there are fewer than 5,000 intensive care beds across 43 countries and less than 2,000 ventilators across 41 countries [19, 20]. Centralized COVID-19 testing, stockouts and unavailability of routine infection prevention and control (IPC) supplies such as masks, gloves, and soap, and access to basic water, sanitation, and hygiene infrastructure further constrain African countries, including Sierra Leone, to effectively detect and respond to this public health threat [21–23].

Sierra Leone documented its first case of COVID-19 in late March 2020 and subsequently implemented several government-led containment measures including a temporary interdistrict lockdown, mandatory 14-day quarantine for international travelers, and isolation and daily monitoring of confirmed COVID-19 cases at established treatment centers. During the 2014–2016 West African Ebola outbreak, the establishment and conduct of daily briefings carried out at Sierra Leone’s national emergency operations center (EOC) were critical to the coordination of the country’s Ebola response [24, 25]. The EOC was the hub for data gathering, priority setting, and iterative response planning [24]. The government of Sierra Leone has built on lessons learned through Ebola and, after declaring COVID-19 a national public health emergency, rapidly formed the national COVID-19 EOC, a taskforce to coordinate the COVID-19 response across government ministries and non-governmental organizations [26]. Expansion of COVID-19 testing and enhanced community surveillance of suspected and confirmed cases have further strengthened the country’s emergency response. Despite containment efforts, COVID-19 cases have steadily increased and there is suspected ongoing community transmission. As of August 3, 2020, Sierra Leone had reported 1,843 COVID-19 cases and 67 deaths [27].

### Assessment objectives

To assess COVID-19 preparedness and inform site interventions to ensure continuity of HIV services, ICAP at Columbia University, in collaboration with the Sierra Leone Ministry of Health and Sanitation (MoHS), conducted rapid site readiness assessments at HFs and community service points (CSPs) providing HIV services in Freetown, Sierra Leone, the epicenter of COVID-19 in the country [28]. In this manuscript, we present findings from this assessment, one of the first HF-level COVID-19 preparedness assessments in West Africa. Although these assessments were driven by the goal of maintaining HIV services during the COVID-19 pandemic, findings may inform maintenance of all essential healthcare services.

## Materials and methods

Assessments were conducted as part of routine service delivery under ICAP’s Resilient and Responsive Health Systems (RRHS) initiative, a technical assistance project aimed to improve HIV service provision and health outcomes at high-volume HIV sites. Data were collected at nine public HFs (five hospitals and four community health centers) and seven CSPs in April 2020, a month after COVID-19 was declared a pandemic. All sites included in the assessment were supported under the RRHS initiative. Among sites, four were in Western Area Rural (2/9 HFs; 2/7 CSPs), and all others were in Western Area Urban. HFs served a mean catchment area of 29,962 people (range: 6,960–52,800) [29]. While the sample represents a convenience sample, these sites have large catchment areas and serve the majority of PLHIV residing in Western Area Urban and Western Area Rural. CSPs comprised three community drop-in centers providing HIV counseling and testing services as well as HIV prevention services (e.g., condoms and lubricants) for key and priority populations, including men who have sex with men, female sex workers, and people who inject drugs, and four community-based support groups serving PLHIV. At the time of the assessment, CSPs did not provide clinical care or ART services but had established mechanisms for linkage to ART with assessed HFs. CSPs were considered potential sites for expansion of differentiated HIV service delivery (DSD) including consideration of using CSPs as ART distribution sites if the healthcare system were overwhelmed in the context of COVID-19. DSD—strategies to improve patient-centered care—aims to minimize challenges in adherence and retention in care through measures such as multi-month dispensing (MMD) and community ART distribution.

The assessment tool was adapted from the Pan American Health Organization Hospital Readiness Checklist for COVID-19 [30], one of the only published COVID-19 preparedness tools at the time of the assessment, to the Sierra Leonean context in collaboration with MoHS. The tool included measures under eight pandemic response function domains: leadership, coordination, health information, rapid identification, diagnosis, isolation, case management (clinical protocols), and IPC. An additional domain related to HIV service delivery in a public health emergency was added and included measures assessing DSD, including MMD and ART stock supply. As CSPs included some sites in nascent stages of healthcare service delivery, CSP-specific data were only collected for applicable measures. Some measures were not collected for one HF as that HF was not designated to support COVID-19 diagnosis in Sierra Leone.

During single site visits, paper-based tools were completed by ICAP and MoHS staff, in consultation with site supervisory staff. Assessments were conducted in English and data were subsequently entered into an Excel database. Descriptive statistics were generated for COVID-19 response function measures by site type. Sites that “met” a response readiness activity were classified as having established a response measure. Sites that “did not meet” or were “in process” of meeting a response readiness activity were classified as not having a measure in place. The Sierra Leone Ethics and Scientific Review Committee deemed all data collected through HF and CSP assessments to be non-human subjects’ research and granted ICAP and MoHS a waiver of ethical review.

## Results

Under the leadership domain, most HFs had staff assigned for IPC (8/9), but lacked staff assigned for COVID-19 screening and triaging (4/9), clinical protocols (3/9), and laboratory activities including sample collection and transfer (2/9) (Table 1). Only 4/9 HFs had a COVID-19 preparedness plan in place. Across CSPs, preparedness was low with just 1/4 CSPs with staff assigned for IPC and none with staff assigned for COVID-19 screening and triaging (0/4), clinical protocols (0/3), and laboratory activities including sample collection and transfer (0/4). Just 4/9 of HFs and 1/4 of CSPs had an established mechanism for the wellbeing and safety of staff during the COVID-19 response.

**Table 1:** COVID-19 preparedness measures by domain and site type.

Domain*	Measure*	HF	CSP
		n = 9	n = 7
Leadership	Committee for COVID-19 emergency response	4/9	2/6
	Designated person responsible for coordinating COVID-19 response activities	5/9	3/6
	COVID-19 preparedness plan in place	4/9	3/6
	Established space (well-equipped) for holding COVID-19 emergency response meetings	5/9	3/6
	Staff assigned for COVID-19 screening and triaging	4/9	0/4
	Staff assigned for IPC	8/9	1/4
	Staff assigned for COVID-19 case management/clinical protocols	3/9	0/3
	Assigned roles and responsibilities for laboratory (sample collection and transfer) activities	2/9	0/4
	Established mechanisms for well-being and safety of personnel during the COVID-19 response, including monitoring of exposed personnel	4/9	1/4
	Mechanism to distribute information on COVID-19 to all staff	7/9	3/5
Coordination	Designated person to liaise with the Emergency Operations Center	7/9	2/6
	Designated person to liaise with treatment centers	2/9	0/5
	Designated person responsible for stock management including medication, PPE, and supplies	8/9	1/5
	Designated person responsible for COVID-19 patient care and transfer	3/9	0/5
Health information	SOPs to collect and validate data and information related to COVID-19	2/9	0/4
	HCWs available to collect, validate data, and information related to COVID-19	4/9	0/4
	Standardized forms for reporting on COVID-19 hospitalizations (including critical care), incidence of suspected and confirmed cases, clinical situation, and deaths	3/9	0/4
	Standardized forms for reporting on COVID-19 screening and triage	2/9	0/4
	Standardized forms for reporting on COVID 19 case-based reporting	1/9	0/4
	Standardized forms for reporting on COVID-19 hospitalization and monitoring	2/9	0/4
Rapid identification	Triage procedure at site entrance, focusing on screening of clients for COVID-19	2/9	0/4
	Trained HCWs for accurate screening, triaging, and reporting of suspected cases	4/9	0/4
	Communications and monitoring system in place for timely alerts and reporting of suspected cases in any area of the site	8/9	1/4
Diagnosis	Established procedures for collecting, handling, and transporting samples to testing centers, following biosafety measures	2/8	0/3
	Trained staff for collecting, handling, and transporting samples to testing centers	1/8	0/3
	Adequate kits in the facility laboratory to collect and package COVID-19 samples	0/8	0/3
	Adequate PPE in laboratory for the handling of samples and disposal of biological waste	2/9	0/3
Isolation unit	Isolation unit for suspected and confirmed cases	3/9	2/7
	Isolation unit equipped+ for medical care of suspected and confirmed COVID-19 cases	1/9	0/4
	Isolation unit equipped with beds for suspected and confirmed COVID-19 cases	3/9	0/4
	Procedures (review, update, and test) for transferring COVID-19 patients to treatment centers	2/9	0/4
	Donning and doffing posters (or job aids) available in PPE areas	2/9	0/5
	Hand hygiene stations available in PPE donning and doffing areas	3/9	1/5
Case management/clinical care	Protocol for management of suspected or confirmed COVID-19 cases	1/9	0/3
	Medical equipment (e.g., oxygen concentrator or ventilator) for initial medical care of suspected/confirmed COVID-19 cases with severe respiratory symptoms in isolation units	1/9	0/3
	Treatment packages per national protocol for suspected or confirmed COVID-19 cases	0/8	0/3
	HCWs trained in the initial management of suspected or confirmed COVID-19 cases	2/9	0/3
	HCWs trained in the continuous management of suspected or confirmed COVID-19 cases	2/9	0/3
Infection prevention and control	Plan in place for protecting patients, healthcare personnel, and visitors from COVID-19	5/9	2/4
	Triage procedures in place for isolation of suspected COVID-19 cases	4/9	1/5
	Design, patient flow and triage system complying with national infection control guidelines	5/9	0/4
	Observe spatial separation of at least 1.5–2 meters between all patients	8/9	5/6
	Waiting area which is well ventilated (i.e. windows kept open, especially in waiting rooms and in-patient settings)	9/9	6/6
	Has IPC checklist	6/9	0/6
	Has an IPC focal person	9/9	1/6
	Procedures (updated and tested) for receiving and transferring COVID-19 patients to treatment centers	5/9	0/5
	HCWs have access to surgical masks for management of suspected/confirmed COVID-19 cases	5/9	2/7
	HCWs have access to face shields for management of suspected/confirmed COVID-19 cases	4/9	0/7
	HCWs have access to gloves for management of suspected/confirmed COVID-19 cases	7/9	2/7
	HCWs have access to gowns/aprons for management of suspected/confirmed COVID-19 cases	4/9	0/7
	HCWs trained in the use of PPE and on additional precautions for specific COVID-19 transmission mechanisms (droplets, contact, aerosols, and fomites)	5/9	0/6
	Protocols or procedures available for cleaning clinical areas, including training in the use of decontamination materials	5/9	0/6
	Protocols for disinfection and sterilization of biomedical equipment and material devices	7/9	1/4
	Area for disinfection and sterilization of biomedical equipment and material devices	9/9	2/5
	Protocol and a marked route for management and final disposal of infectious biological waste, including sharps	8/9	1/6
	Infrastructure and procedures for proper hand hygiene, including handwashing, continuous training, and supplies	7/9	4/6
	Guidelines for handling of bodies of those deceased from COVID-19	2/9	0/3
	Routine cleaning of clinical ward outside the patient’s room	8/9	2/5
	HCWs trained on safety management and disposal of COVID-19 contaminated materials	3/9	0/3
HIV services in public health emergencies	SOPs or guidelines for providing DSD during public health emergencies	3/9	2/4
	HCWs trained in SOPs or guidelines for providing DSD during public health emergencies	2/9	1/3
	Standard tools for documenting DSD during public health emergencies	3/8	1/3
	HCWs trained on tools for documenting DSD during public health emergencies	3/9	0/3
	Adequate ARVs in stock for MMD for all PLHIVs	7/9	0/3

Acronyms: ARVs: antiretrovirals, DSD: differentiated HIV service delivery, HCWs: healthcare workers, IPC: infection prevention and control, PLHIV: people living with HIV, PPE: personal protective equipment, SOPs: standard operating procedures.

+Equipped with oxygen concentrator, thermometer, blood pressure monitor, and pulse oximeter.

*Domains and measures structured according to an adapted version of the Pan American Health Organization Hospital Readiness Checklist for COVID-19.

While most HFs had a designated person to liaise with the national EOC (7/9) and manage stock including personal protective equipment (PPE) (8/9), coordination with treatment centers (2/9) and for COVID-19 patient care and transfer (3/9) was limited among HFs and nonexistent for CSPs (0/5). Only one CSP had a designated person responsible for stock management including PPE (1/5).

Health information measures were also low. HFs lacked standardized forms for reporting on COVID-19 screening and triage (2/9), COVID-19 case-based reporting (1/9), and COVID-19 hospitalization and monitoring (2/9). Few HFs had HCWs available to collect and validate COVID-19-related data (4/9) and SOPs on COVID-19 data collection (2/9). No CSPs had any measure under the health information domain in place (0/4).

Few sites had a triage procedure at the site entrance focused on screening clients for COVID-19 (2/9 HFs; 0/4 CSPs) and had trained HCWs for accurate screening, triaging, and reporting of suspected cases (4/9 HFs; 0/4 CSPs). While HFs had a communications and monitoring system for timely alerts and reporting of suspected cases (8/9), most CSPs did not (1/4). Diagnosis measures were also low. HFs lacked protocols (2/8) and staff for collecting and transporting samples (1/8), adequate kits to collect and package samples (0/8), and adequate PPE for handling samples and waste disposal (2/9).

Moreover, few HFs had isolation units for suspected or confirmed cases (3/9), particularly isolation units equipped for medical care (1/9). Hand hygiene stations available in PPE donning and doffing areas (3/9) and job aid availability in these areas were limited among HFs (2/9). Among CSPs, 2/7 had an isolation unit though none had equipment for medical care (0/4) or beds (0/4). Hand hygiene stations were also unavailable in PPE donning and doffing areas among most CSPs (1/5).

Few HFs had a protocol for initial or ongoing management of suspected or confirmed COVID-19 cases (1/9), medical equipment (e.g., oxygen concentrator or ventilator) (1/9), or HCWs trained in initial management (2/9) or ongoing management of suspected or confirmed COVID-19 cases (2/9). Of the three CSPs providing HIV prevention and counseling and testing services, none had measures under this domain in place.

Under the IPC domain, more than half of HFs had trained HCWs on the use of PPE and precautions to prevent COVID-19 transmission (5/9) though fewer had trained HCWs on safe management and disposal of COVID-19 contained materials (3/9). HCWs at most HFs had access to surgical masks (5/9) and gloves (7/9) to manage suspected/confirmed COVID-19 cases and 7/9 had infrastructure for proper hand hygiene. Conversely, few CSPs had access to surgical masks (2/7), gloves (2/7), or gowns/aprons (0/7) for management of suspected/confirmed COVID-19 cases, and no HCWs had been trained in the use of PPE and precautions to prevent COVID-19 transmission (0/6). While waiting areas were well ventilated across sites (9/9 HFs; 6/6 CSPs), fewer HFs (5/9) and CSPs (0/4) had a site design and patient flow that complied with national IPC guidelines. Only 4/9 HFs and 1/5 CSPs had a triage procedure for isolation of suspected COVID-19 cases. Appropriate protocols for cleaning clinical areas were limited (5/9 HFs; 0/6 CSPs).

When assessing HFs’ ability to provide HIV services in a public health emergency, 7/9 had adequate stock of antiretrovirals (ARVs) for MMD though few had procedures for providing DSD (3/9) and had trained HCWs on providing DSD (2/9). Among CSPs, 2/4 had procedures for providing DSD, 1/3 had HCWs trained on procedures and none had adequate stock of ARVs (0/3).

## Discussion

Taken together, these findings highlight gaps across COVID-19 preparedness domains at high-volume HIV sites serving PLHIV in Sierra Leone one month after COVID-19 was declared a pandemic. Substantial gaps in COVID-19 leadership, coordination, health information, rapid identification, diagnosis, isolation, clinical procedures, and COVID-specific IPC measures were documented, despite significant investments in global health security measures in Sierra Leone during and after the Ebola outbreak, including expansion of infectious disease surveillance, investments in public health infrastructure and supplies, and IPC training at various levels of the health system.

While findings highlight universal gaps across site types, given distinct needs between site types, in these high-volume HIV sites, CSPs may require more intensive supportive supervision and training to ensure HIV services are uninterrupted while minimizing COVID-19 risk, especially if used as sites to scale up DSD. Acute differences by site type, particularly in IPC, may be a result of interventions aimed at HFs during the Ebola outbreak. Most staff at assessed HFs received training in IPC during the Ebola outbreak. HFs had some IPC measures in place in response to Ebola, including an IPC focal person and infrastructure to support IPC (spatial separation of patients, well ventilated waiting areas, handwashing stations and supplies, and areas for disinfection of equipment). In contrast, CSPs lacked most IPC measures.

Identification of gaps in preparedness is a critical first step in providing needed training and support for COVID-19 preparedness. Using results from this assessment, ICAP, in collaboration with MoHS, developed site-specific supportive supervision and training plans to ensure HIV services continue while minimizing COVID-19 risk. COVID-19 support included the establishment of triage centers to screen patients for COVID-19 symptoms, HCW training in COVID-19-specific IPC measures, guidelines, reporting, and protocols, provision of IPC supplies, and site-specific support to ensure domain response function measures are in place. To decongest HFs and reduce potential exposure to COVID-19 for patients and HCWs, MoHS with ICAP support, accelerated implementation of MMD for all PLHIV starting in May 2020 and minimized the need for face-to-face care by pivoting to a model based on routine telephone contact with PLHIV [31, 32]. Sierra Leone has shown that amidst a public health crisis, innovations to advance HIV service delivery, such as DSD and telemedicine can be expedited. Gaps in preparedness under the HIV service delivery domain informed site-specific interventions, including HCW training and mentoring, supportive supervision in DSD guidelines, and use of DSD tools as well as distribution of DSD-specific SOPs and tools. For CSPs, the assessment provided needed information on preparedness domains requiring immediate focus if DSD scale up were to include CSP provision of MMD refills and patient management.

Sierra Leone continues to face a massive HCW shortage. COVID-19 infection among HCWs, as well as HCW strikes threaten Sierra Leone’s already fragile health system. As was done during the Ebola outbreak [33], HCWs in Sierra Leone and elsewhere in Africa have suspended care for patients at some HFs over unpaid hazard or bonus payments and insufficient supply of PPE [33–35] as occupational risks of COVID-19 infection includes lack of or improper use of PPE and sub-optimal adherence to IPC measures [36]. HCW strikes, infection and mortality, coupled with psychosocial hazards such as witnessing higher suffering and mortality, longer or irregular hours, and higher workload, paints an eerily familiar picture to experiences during Ebola [37]. Since losing 7% of the health workforce due to death during Ebola [2, 3], Sierra Leone has attempted to rebuild and strengthen the health workforce. While estimates may be higher due to underreporting and gaps in COVID-19 testing, approximately 172 HCWs have been reported as infected with COVID-19 in Sierra Leone since the outbreak (9% of total cases) [38], presenting risks of further deterioration to the health workforce.

This assessment has limitations, including that many measures were not applicable for the CSPs because the original tool was developed for HFs. Applicability of measures was determined by ICAP or MoHS staff collecting data, in collaboration with the site. As data were urgently needed to inform site support during a public health emergency, the tool was not piloted in Sierra Leone prior to use. This assessment did not include a domain on psychosocial preparedness; given higher suffering and mortality and potential occupational risk of COVID-19 infection for HCWs, as was documented during Ebola [39, 40], future preparedness assessments should include measures to assess this domain. As sites included in the assessment represent a convenience sample of selected high-volume HIV sites, these findings may not be generalizable to other care sites. Despite these limitations, this rapid assessment provided important information on needed COVID-19 and HIV training and support at sites serving a large PLHIV population in Sierra Leone, which allowed MoHS and ICAP to quickly address gaps in preparedness across COVID-19 response function domains.

## Conclusions

Findings from this assessment highlighted gaps across COVID-19 preparedness domains at sites serving PLHIV in Freetown, Sierra Leone, the epicenter of the COVID-19 epidemic in the country. Identification of gaps in COVID-19 preparedness is a critical step in providing needed support for infection control and modified service delivery. In these high-volume HIV sites, CSPs may require more intensive supervision and training than HF to ensure HIV services are uninterrupted, especially if used as sites to scale up DSD.
